# Evaluation and Anomaly Detection Methods for Broadcast Ephemeris Time Series in the BeiDou Navigation Satellite System

**DOI:** 10.3390/s24248003

**Published:** 2024-12-14

**Authors:** Jiawei Cai, Jianwen Li, Shengda Xie, Hao Jin

**Affiliations:** School of Information and Electronic Engineering, Zhejiang University of Science and Technology, Liuxia Street, Hangzhou 310023, China; jiawei.cai@zust.edu.cn (J.C.); shengda.xie@zust.edu.cn (S.X.); hao.jin@zust.edu.cn (H.J.)

**Keywords:** BDS, broadcast ephemeris, threshold, machine learning, anomaly detection, time-series prediction

## Abstract

Broadcast ephemeris data are essential for the precision and reliability of the BeiDou Navigation Satellite System (BDS) but are highly susceptible to anomalies caused by various interference factors, such as ionospheric and tropospheric effects, solar radiation pressure, and satellite clock biases. Traditional threshold-based methods and manual review processes are often insufficient for detecting these complex anomalies, especially considering the distinct characteristics of different satellite types. To address these limitations, this study proposes an automated anomaly detection method using the IF-TEA-LSTM model. By transforming broadcast ephemeris data into multivariate time series and integrating anomaly score sequences, the model enhances detection robustness through data integrity assessments and stationarity tests. Evaluation results show that the IF-TEA-LSTM model reduces the RMSE by up to 20.80% for orbital parameters and improves clock deviation prediction accuracy for MEO satellites by 68.37% in short-term forecasts, outperforming baseline models. This method significantly enhances anomaly detection accuracy across GEO, IGSO, and MEO satellite orbits, demonstrating its superiority in long-term data processing and its capacity to improve the reliability of satellite operations within the BDS.

## 1. Introduction

The development of Global Navigation Satellite Systems (GNSSs) has greatly advanced satellite-based Positioning, Navigation, and Timing (PNT) applications [1,2,3]. However, anomalies in broadcast ephemeris data caused by hardware malfunctions, software errors, or orbital adjustments can degrade positioning accuracy [4]. Therefore, ensuring reliable GNSS performance requires effective anomaly detection. Traditional detection methods, which mainly rely on physical models [5], threshold-based techniques, and Signal-in-Space Range Error (SISRE) calculations [6,7], are often limited in their ability to process complex data. In contrast, deep learning methods have shown superior efficacy in feature extraction, enhancing both real-time capability and anomaly detection accuracy. Although research on deep learning for BDS anomaly detection is still in its infancy, it holds significant potential to improve the reliability of satellite navigation systems [8,9].

Conventional threshold- and model-based methods typically detect anomalies by comparing variations in ephemeris parameters to predefined thresholds. These methods are simple and provide clear physical interpretations [10,11,12]. Remondi (1989) introduced threshold ranges for orbital parameters by comparing GPS broadcast ephemeris with precise ephemeris data [13], while Liu Chaoying et al. proposed innovative techniques, including gross error detection, ephemeris consistency checks, and a pseudorange OMC median-based method, which have proven highly effective [14]. However, single-threshold approaches often fail to capture all potential anomaly scenarios, lack adaptability to dynamic system changes, and suffer from suboptimal real-time responsiveness. As a result, recent research increasingly favors hybrid methods to improve detection performance and adaptability.

To address these limitations, SIS-based methods have gained prominence, particularly due to the growing demand for high-accuracy satellite signals. Gao et al. introduced two SIS error calculation methods: the ‘top-down’ and ‘bottom-up’ approaches [15]. The ‘bottom-up’ approach, relying on receiver data, uses geometric analysis and redundancy checks to monitor navigation signal integrity, exemplified by Receiver Autonomous Integrity Monitoring (RAIM) [16]. This technique effectively identifies ephemeris anomalies through multi-observation strategies, such as pseudorange and carrier-phase observations. Techniques like Recursive Least Squares and Kalman Filtering further improve pseudorange detection accuracy. In contrast, the ‘top-down’ approach uses ground control station data to correct non-spatial signal errors and calculates SIS errors via satellite laser ranging and ground monitoring station data, which are widely applied in multi-constellation systems for SISRE assessments [17].

In contrast to traditional techniques, which are constrained by human expertise and single-model approaches, the rapid advancements in machine learning [18] have generated significant interest in its use for broadcast ephemeris anomaly detection. Machine learning can identify complex correlations in historical data, improving detection accuracy and adaptability. Han et al. [19] applied machine learning to analyze ephemeris data and track TEC variations resulting from varying LEO altitudes, developing a three-dimensional TEC model using LEO data. Other studies have explored pseudorange and phase observations through feature extraction and regression modeling, assessing GNSS positioning performance across different temporal and data dimensions using machine learning. A comparative analysis of the Decision Tree (DT), Random Forest (RF), and Support Vector Machine (SVM) models showed that RF regression outperformed the other models [20]. Additionally, LSTM and GRU models have been used to mitigate orbital and clock errors in GPS, demonstrating that these errors are not random but can be effectively modeled [21]. These findings underscore the ability of machine learning to address the limitations of traditional methods, providing significant advantages in real-time processing and in managing system complexities.

Although deep learning holds promising potential for broadcast ephemeris anomaly detection, research in this field remains limited. This study introduces an automated anomaly detection framework based on the IF-TEA-LSTM model and big data analytics. The original dataset is organized as a multivariate time series, followed by feature analysis, data integrity assessment, stationarity testing, differencing, and deliberate anomaly introduction via SIS. The IF-TEA-LSTM model is applied to perform a comprehensive analysis of threshold sensitivities, mean square errors, and prediction accuracies across different satellites. This approach facilitates the detailed classification of clock errors, orbital parameters, and five key orbital elements, along with an extensive analysis of system features across various satellite types. The proposed model shows significant improvements over traditional methods, addressing challenges in long-cycle data processing, enhancing threshold sensitivity, improving detection efficiency, and exhibiting strong resilience to adversarial perturbations.

## 2. Research Methods

This study focuses on the implementation of anomaly detection techniques using BDS broadcast ephemeris data. First, we introduce an enhanced iForest algorithm to extract anomaly score features. Subsequently, we present the IF-TEA-LSTM method, which is employed to predict broadcast ephemeris data and detect anomalies by comparing the predicted values with the actual data.

### 2.1. Enhanced iForest Anomaly Scoring Method

iForest is an anomaly detection algorithm that uses isolation trees to detect anomalies. It identifies anomalies by constructing multiple isolation trees and calculating anomaly scores based on the path lengths within the trees.

iForest [22] recursively constructs isolation trees to detect anomalies. By randomly selecting a feature and a split value, the algorithm partitions the subsampled data until it reaches a predefined tree depth or node count. Anomalies, due to their distinct behavior in the feature space [23], are typically isolated at shallower levels of the tree. By aggregating results from multiple isolation trees, iForest efficiently identifies and ranks anomalies. The average path length, c(n), of an isolation tree for a dataset of size *n* is standardized using the harmonic number H(i), as shown in Equation (1).

(1) $$c\left(n\right)=2H\left(n-1\right)-\frac{2(n-1)}{n}$$

Anomaly scores are essential for quantifying the degree of anomaly in data points. The anomaly score, s(x,n), for a data point *x* is computed using Equation (2).

(2) $$s\left(x,n\right)=2^{-\frac{E(h(x))}{c(n)}}$$

In iForest, the path length frequently prioritizes global anomalies, complicating the effective isolation of anomalous points, especially in cases of recurring anomalies with high scores. To overcome this challenge, we propose substituting the global ranking based on path length with relative mass metrics [24] and local ranking. This method calculates the local anomaly score $s_{i}\left(x\right)$ for a specific instance *x* by determining the mass ratio between nodes along the path from the root to the leaf node, as articulated in Equation (3).

(3) $$s_{i}\left(x\right)=\frac{m\left(T_{i}\left(x\right)\right)}{m\left(T_{i}\left(x\right)\right)\times \psi }$$

Here, $s_{i}\left(x\right)$ captures local anomalies, differentiating $s_{i}\left(x\right)$ from the global anomaly score, s(x,n). $T_{i}\left(x\right)$ denotes the leaf node, *m* is the node’s mass, and ψ is a normalization factor. The overall anomaly score, S(x), is the average of local anomaly scores across all trees, as formulated in Equation (4).

(4) $$S\left(x\right)=\frac{1}{t}\sum_{i=1}^{t}s_{i}\left(x\right)$$

The relative mass, given by Equation (5), defines $m_{p}$ as the parent node mass, $m_{l}$ as the child node mass, and ψ as the normalization term. This calculation improves anomaly detection by quantifying the relative mass of samples, enhancing sensitivity to anomalies in both sparse and dense regions.

(5) $$\mathrm{RM}\left(m_{p},m_{1},\psi \right)=\frac{m_{1}}{m_{p}\times \psi }$$

Unlike previous studies that focused solely on relative mass calculations, this paper presents a hybrid approach that combines relative mass with path length. Specifically, the total path length to the leaf node is integrated with the parent–child mass ratio, incorporating a penalty mechanism, as illustrated in Equation (6).

(6) $${\mathrm{RM}}_{2}=d+\frac{\log_{2}\left(p\right)}{\omega \times \log_{2}\left(l\right)}+log_{2}\left(\frac{p}{l}\right)$$

In this equation, *d* represents the depth of the leaf node, while *l* and *p* denote the number of instances in the leaf and parent nodes, respectively. The depth reflects the hierarchical significance of mass and serves as a key factor in the anomaly score.

**Figure 1:**
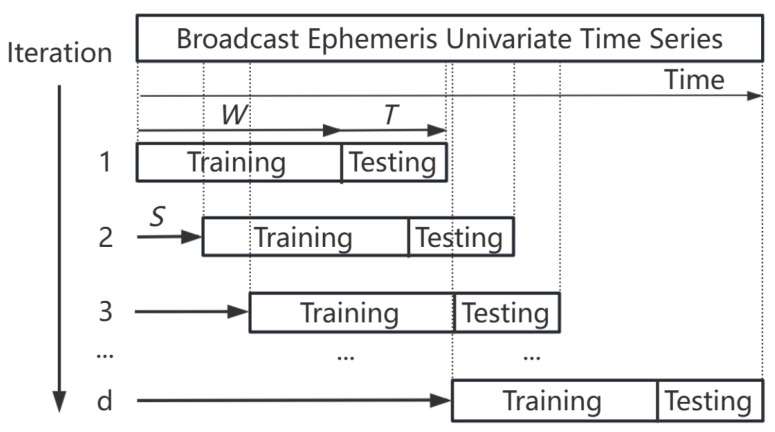
Schematic diagram of incremental updates for window scrolling.

Traditional iForest methods may lose essential features when applied to time-series data, and the complex distribution of broadcast ephemeris time series adds further challenges. In such cases, a single relative mass metric proves insufficient. Therefore, we integrate adjacent point difference detection with multi-scale sliding windows to incrementally update the data stream and achieve balanced anomaly scores over time. The weights are derived from the feature importance of each satellite, as shown in Figure 1.

**Figure 2:**
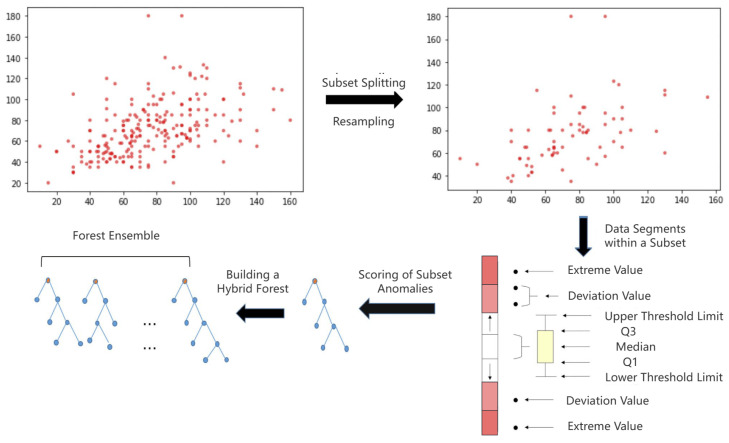
Flowchart for constructing anomaly score forest clusters.

This method divides long time series into subsets based on periodic fluctuations, extracting features using sliding windows. During the iterative process, comprehensive anomaly scores are used to identify outliers. *U* represents the number of available time-series cycles, W+T specifies the window size derived from difference detection, and *d* indicates the number of iterations of the data stream. The final anomaly score stream is depicted in Figure 2.

In the context of broadcast ephemeris data, data sequences with shorter path lengths and feature values that deviate significantly from normal values within a subset (i.e., exhibiting a high relative mass ratio) are more likely to be classified as anomalies. Additionally, a penalty mechanism is introduced for data points that, despite being prominent in the overall dataset, show no significant deviation within the subsets. This mechanism incorporates factors such as orbital maneuvers as cutting points while considering key characteristics of broadcast ephemeris time series, including low anomaly probability, frequent extreme values, and notable feature shifts. Together, these elements enhance the accuracy of anomaly detection scores.

By utilizing neighboring point difference detection, the dataset is partitioned into subsets, and anomaly scores are calculated for each subset. As shown in Figure 2, the box plot illustrates the distribution of data within the normal range. Data points that exceed the threshold are assigned higher anomaly scores, with extreme values receiving the highest scores. The anomaly scores from different subsets are then aggregated to form a forest ensemble, with path lengths applied to the overall dataset and relative mass applied to the subsets, resulting in a hybrid scoring system.

While the forest ensemble captures the relationships among multiple parameters within the satellite, it does not effectively exchange feature information. As a result, the model may mistakenly classify the anomaly scores of randomly selected single-column data as indicators of anomalies, undermining the practical significance of multivariate data. To address this limitation, a deep learning-based time-series prediction model is introduced.

### 2.2. IF-TEA-LSTM

Recurrent Neural Networks (RNNs) [25,26] are effective for modeling sequential data by leveraging temporal dependencies. However, they face challenges, such as vanishing gradients. Long Short-Term Memory (LSTM) networks address these issues with a gating mechanism that includes forget, input, and output gates. This design enables LSTM to effectively capture complex long-term dependencies and temporal patterns. While Gated Recurrent Units (GRUs) [26,27] offer a simplified, computationally efficient alternative by merging certain gates, they lose some of the nuanced control present in LSTM networks. Given the complexity of the data and the need for accurate dependency modeling, LSTM is more appropriate for this study.

Building on LSTM’s strengths, the IF-TEA-LSTM (Improved iForest-based Thresholding and Attention-Augmented Long Short-Term Memory) model introduces several innovations for anomaly detection in time-series data [28,29]. The model consists of three main components: an enhanced data stream derived from the improved iForest (IF) model, a dual-threshold mechanism, and an attention-augmented LSTM architecture. To evaluate the impact of each component, ablation studies are conducted by sequentially removing or modifying these elements, resulting in the LSTM, A-LSTM (Attention-Enhanced LSTM), and TE-LSTM (Threshold-Enhanced LSTM) variants.

**Figure 3:**
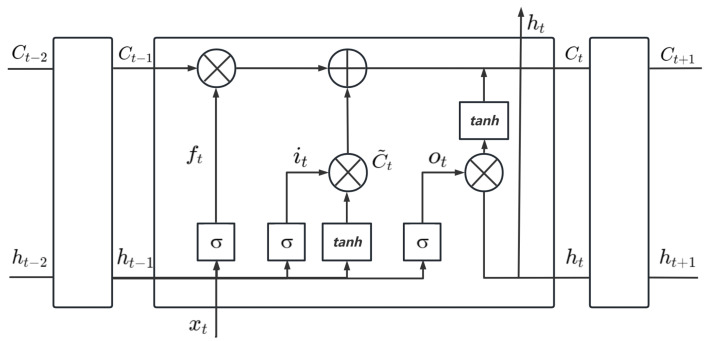
LSTM unit.

The basic LSTM module unit is illustrated in Figure 3.

The flow of information through the LSTM blocks during the forward propagation phase is articulated as shown in Equations (7)–(10).

(7) $$f_{t}=\sigma \left(W_{f}\cdot \left[h_{t-1},x_{t}\right]+b_{f}\right)$$

(8) $$i_{t}=\sigma \left(W_{i}\cdot \left[h_{i-1},x_{t}\right]+b_{i}\right)$$

(9) $$o_{t}=\sigma \left(W_{O}\cdot \left[h_{\iota -1},x_{\iota }\right]+b_{o}\right]$$

(10) $${\tilde{C}}_{t}=tanh\left(W_{C}\cdot \left[h_{t-1},x_{t}\right]+b_{c}\right)$$

In these equations, $i_{t}$, $o_{t}$, and $f_{t}$ represent the input gate, output gate, and forget gate, respectively, while ${\tilde{C}}_{t}$ denotes the candidate value of the current neuron. The matrices $W_{i}$, $W_{O}$, $W_{C}$, and $W_{f}$ correspond to the respective weights applied to the gates and the candidate value, interacting with the contextual inputs, including static features and short-term memory. The biases $b_{f}$, $b_{i}$, $b_{o}$, and $b_{C}$ are their associated bias terms. Additionally, σ represents the activation function.

The new cell state $C_{t}$ is determined by the previous cell state $C_{t-1}$, the forget gate $f_{t}$, the input gate $i_{t}$, and the candidate value ${\tilde{C}}_{t}$. The hidden layer’s output $h_{t}$ is then calculated, as shown in Equations (11) and (12).

(11) $$C_{t}=f_{t}\cdot C_{t-1}+i_{t}\cdot C_{t}$$

(12) $$h_{t}=o_{t}\cdot \tanh\left(C_{t}\right)$$

Building on the principles of LSTM, the IF-TEA-LSTM model adapts these concepts to address the challenges of anomaly detection in time-series data. By combining LSTM’s gated structure with new enhancements, the model effectively captures temporal dependencies and highlights critical features [30,31].

The model uses advanced thresholding techniques to generate weighted anomaly scores. These scores are then fed into the LSTM network as additional inputs, improving the model’s anomaly detection performance. Furthermore, the LSTM network includes an attention mechanism [32,33], which focuses on important time steps. The mechanism calculates an attention score, $score(r,h_{i})$, through a linear transformation and dot product between input hidden states and a target vector, as shown in Equation (13).

(13) $$\mathrm{score}\left(r,h_{i}\right)=r^{T}Wh_{i}$$

where *r* is the query vector, which is used for comparison with the hidden state, *W* is a trainable weight matrix that adjusts and transforms the input vector, and $h_{i}$ is the hidden state vector, with $r^{T}$ representing the transpose of the query vector for the dot-product operation.

Subsequently, the attention scores are normalized across the sequence of outputs from the final LSTM hidden layer. This normalization assigns weights to each time step based on the relative importance of its corresponding hidden state, as shown in Equation (14).

(14) $$\alpha_{i}=\frac{\exp(score\left(r,h_{i}\right))}{\sum_{i}^{j=1}\exp\left(score(r,h_{j})\right)}$$

In this equation, *i* represents the index of the elements in the LSTM hidden layer output sequence, $\exp(\mathrm{score}\left(r,h_{i}\right))$ denotes the importance of the hidden state with a higher LSTM score, and $\alpha_{i}$ indicates the degree of similarity between the element to be encoded in the LSTM hidden layer output sequence and the other elements.

Using these attention weights, a weighted sum of all hidden states is computed to obtain a context vector, as shown in Equation (15).

(15) $$c=\sum_{i=1}^{t}\alpha_{i}h_{i}$$

To reduce the error range in anomaly detection, a thresholding mechanism is implemented to constrain the output of the context vector [34,35]. Specifically, the components of the context vector are limited by an upper threshold, $\theta_{u}$, and a lower threshold, $\theta_{i}$. The penalty term applied to each hidden state component in the context vector is expressed as shown in Equation (16).

(16) $$P\left(c_{i}\right)=\left\{\begin{array}{cc} \lambda {\left(c_{i}-\theta_{u}\right)}^{2}, & \;\mathrm{if}\;\;c_{i}>\theta_{u}\\ \lambda {\left(\theta_{i}-c_{i}\right)}^{2}, & \;\mathrm{if}\;\;c_{i}<\theta_{i}\\ 0, & \;\mathrm{if}\;\;\theta_{i}\leq c_{i}\leq \theta_{u} \end{array}\right.$$

where λ is the penalty coefficient that controls the intensity of the penalty when the values exceed the thresholds.

During training, the total penalty loss is computed by summing the penalty terms across all context vectors. The total penalty loss, $L_{p}$, is given by Equation (17).

(17) $$L_{p}=\sum_{i=1}^{N}P\left(c_{i}\right)$$

(18) $$L_{t}=L_{b}+\alpha L_{p}$$

where *N* represents the dimension *c* of the context vector, excluding the anomaly score and timestamp. This penalty loss is incorporated into the overall loss function, $L_{t}$, which combines the base loss function, $L_{b}$, with the penalty term, as shown in Equation (18).

In the feature fusion-based prediction model, the prediction error at each time step is compared to a predefined threshold. If the error exceeds this threshold, the time step is classified as anomalous; otherwise, it is considered normal. The prediction error, denoted as $e_{t}$, is calculated as shown in Equation (19).

(19) $$e_{t}=\left|y_{t}-{\hat{y}}_{t}\right|$$

where ${\hat{y}}_{t}$ is the predicted value and $y_{t}$ is the true data value.

**Figure 4:**
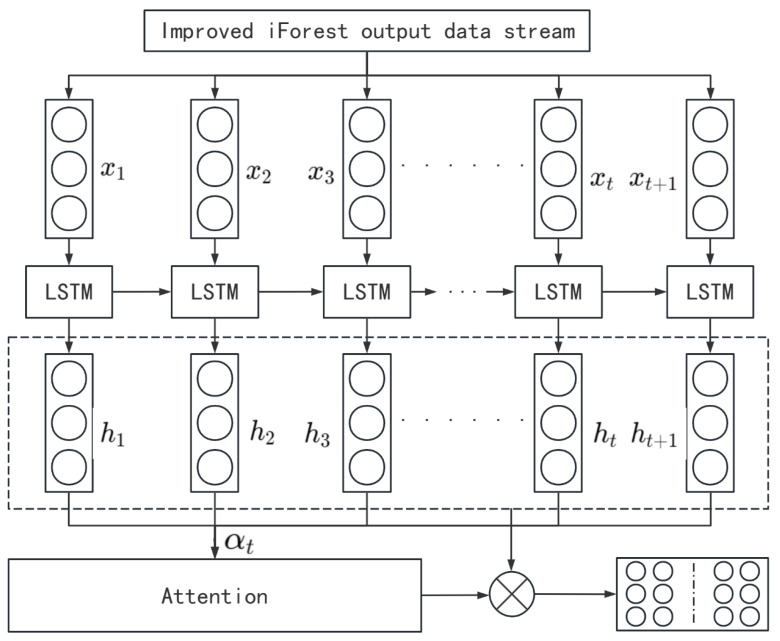
LSTM.

As illustrated in Figure 4, the LSTM network processes the IF-filtered input sequence $x=x_{1},x_{2},\ldots ,x_{n}$, producing hidden states $h=h_{1},h_{2},\ldots ,h_{n}$ that capture temporal patterns. At each time step, the cell state $C_{t}$ is updated to retain critical information. A self-attention mechanism assigns weights $\alpha_{t}$ to the hidden states, emphasizing their importance in the prediction. The weighted sum of $h_{t}$ generates a context vector that highlights key features, enhancing prediction accuracy.

**Figure 5:**
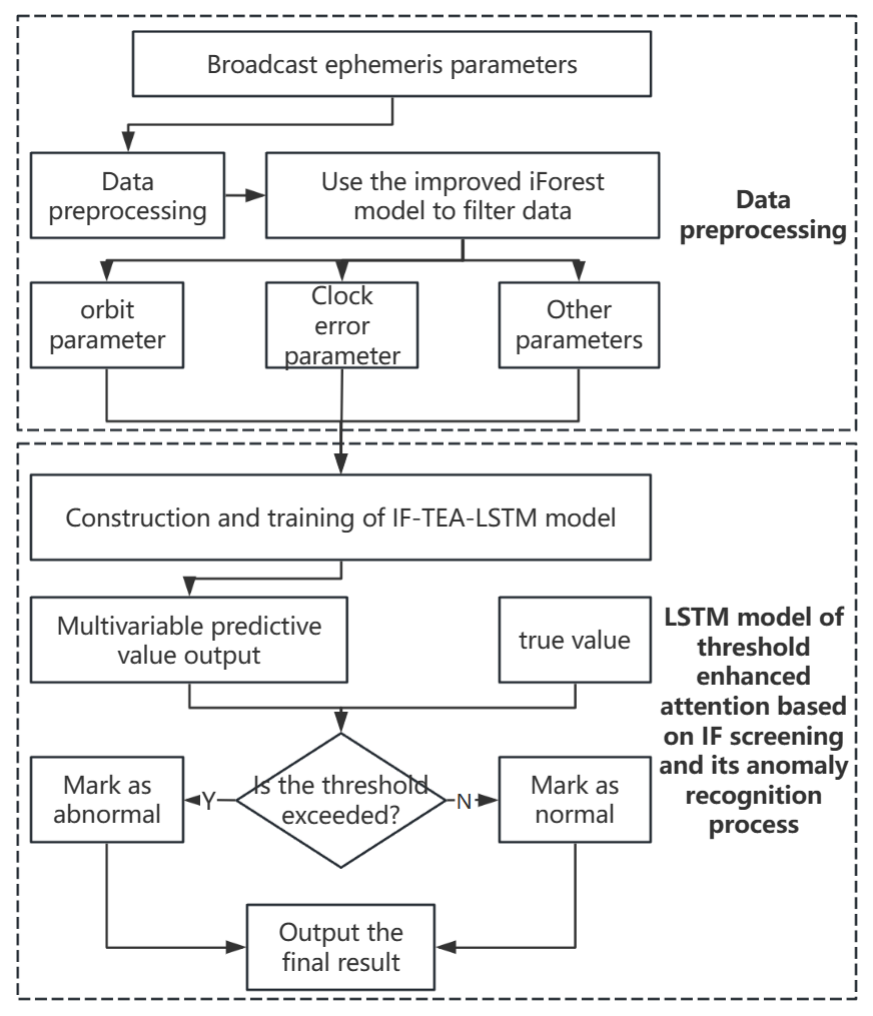
Anomaly detection framework based on IF-TEA-LSTM.

The integration of LSTM with self-attention strengthens temporal representation and significantly improves anomaly detection in multivariate time series. As depicted in Figure 5, the attention-guided framework and thresholding mechanism enhance the reliability and accuracy of BDS broadcast ephemeris data analysis.

## 3. Preparation and Evaluation of BDS Broadcast Ephemeris Experimental Data

### 3.1. Data Conversion

**Figure 6:**
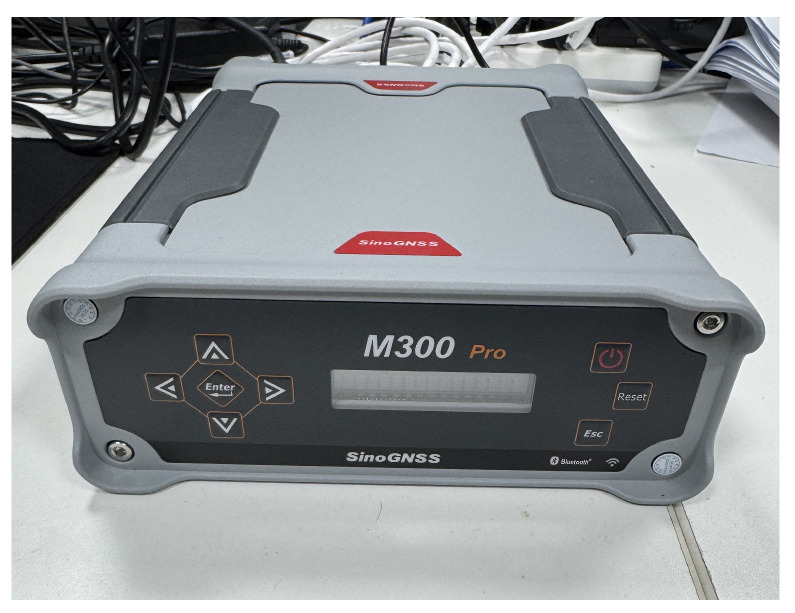
M300 RPO receiver main unit.

**Figure 7:**
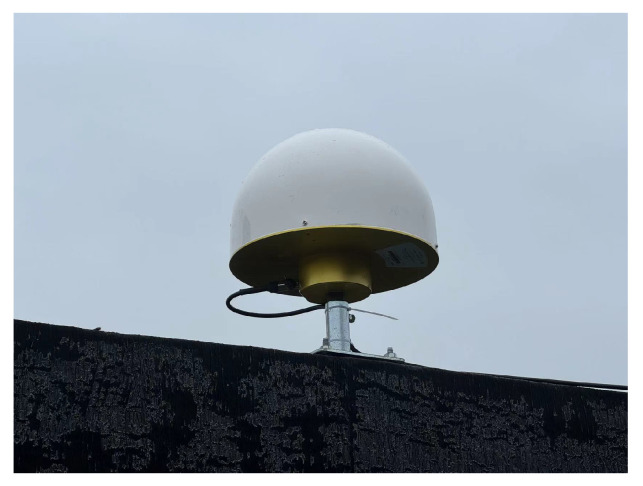
M300 RPO receiver antenna.

Before converting the BDS broadcast ephemeris data into a usable CSV format, RTKLIB [36] was employed to process RINEX .n files obtained from both the receivers and the data center. Schematic diagrams of the M300 PRO’s receiver and antenna are shown in Figure 6 and Figure 7. Subsequently, a database was established to enable RTKLIB to define the data as callable files for efficient extraction. Finally, the extracted data were processed to meet the requirements for the subsequent analyses.

The data extracted from the receiver and the broadcast ephemeris in RINEX version 3.04 can be organized into a time series comprising four major categories [37]. These include the Keplerian parameters: $\sqrt{A},e,i_{0},M_{0},\omega ,\Omega_{0}$; the spherical harmonic coefficients: $c_{rc},c_{rs},c_{uc},c_{us},c_{ic},c_{is}$; the clock correction parameters: $af_{0},af_{1},af_{2}$; and additional parameters: $t_{oe},\Delta n,\mathrm{IDOT},\dot{\Omega }$. Each parameter possesses distinct physical significance and exhibits clear interrelations, as dictated by established dynamic models. The corresponding equation is presented in Equation (20).

(20) $$\begin{array}{ll} C_{1-20} & =[C_{1},C_{2},...,C_{19},C_{20}]\\ \; & =\{\mathrm{date},t_{\mathrm{oc}},\sqrt{A},e,i_{0},\Omega ,\omega ,M_{0},\Delta n,\dot{\Omega },\mathrm{IDOT},\\ \; & \;C_{rc},C_{rs},C_{uc},C_{us},C_{ic},C_{is},af_{0},af_{1},af_{2}\} \end{array}$$

### 3.2. Data Sources

This study primarily utilized data sourced from the Wuhan University IGS Data Center, with gaps supplemented by data from NASA’s CDDIS. The dataset spans the period from 1 January 2021 to 31 December 2021. Table 1 provides a detailed summary of the satellite data acquisition sources, including the data type, time span, and FTP addresses for both the Wuhan University IGS Data Center and CDDIS. During data processing, RTKLIB was employed for data retrieval, with TOC used as the reference for conversion to a standardized time format.

### 3.3. Data Statistical Analysis

This section examines six representative satellites from the BDS2 and BDS3 constellations, spanning the GEO, IGSO, and MEO orbital categories. Specifically, C01 corresponds to BDS2-GEO, C14 corresponds to BDS2-MEO, C16 corresponds to BDS2-IGSO, C26 corresponds to BDS3-MEO, C38 corresponds to BDS3-IGSO, and C60 corresponds to BDS3-GEO. The broadcast ephemeris data display distinct characteristics, often appearing in correlated pairs governed by physical equations, revealing complex interdependencies. These parameters also exhibit periodicity, variability, and threshold behaviors that indicate anomalies such as jumps and maneuvers. To visualize these properties, this study analyzes 15 ephemeris parameters from the six satellites, sampled at one-hour intervals, providing a comprehensive and intuitive depiction of their variation patterns.

**Figure 8:**
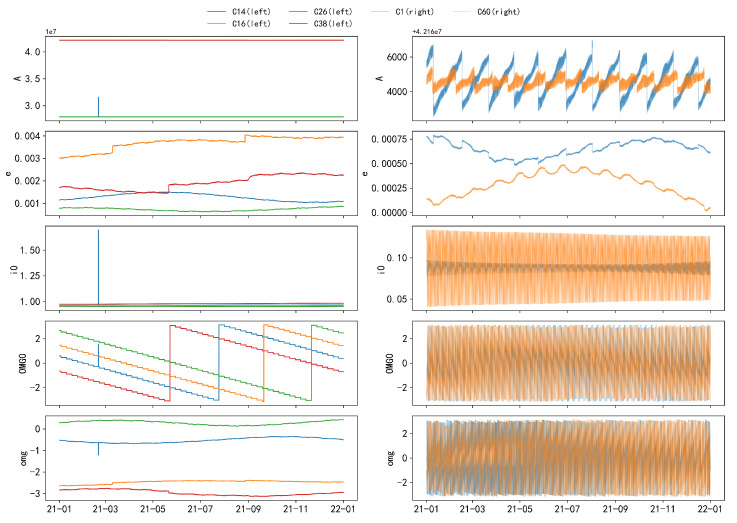
Comparative analysis of the five parameter sets ($\sqrt{A}$, *e*, $i_{0}$, Ω, ω) for MEO and IGSO orbits, along with GEO orbit parameters, based on hourly sampling. The five subplots on the left, from top to bottom, represent the parameters $\sqrt{A}$, *e*, $i_{0}$, Ω, and ω for MEO and IGSO orbits, while the right side corresponds to GEO. The differences between the two orbit types are prominently highlighted.

**Figure 9:**
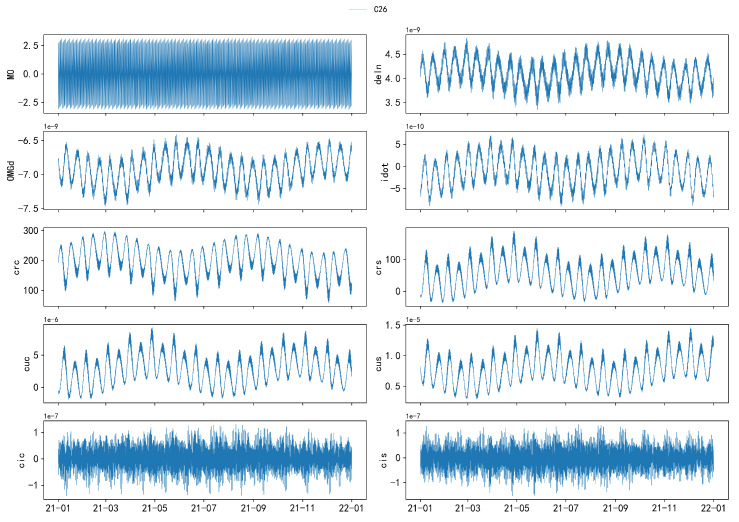
Visualization of the distribution of 10 broadcast ephemeris parameters for the C26 satellite with hourly sampling. From top to bottom, left to right, the parameters are $M_{0}$, Δn, $\dot{\Omega }$, IDOT, $C_{rc}$, $C_{rs}$, $C_{uc}$, $C_{us}$, $C_{ic}$, and $C_{is}$.

The following figures illustrate the time series of orbital parameters from $C_{3}$ to $C_{17}$, as described in Equation (20). Due to significant differences between GEO and IGSO/MEO satellites, a comparative analysis is presented in Figure 8 to highlight these distinctive characteristics. For the remaining parameters, which exhibit similar patterns, C26 is selected as a representative example for further illustration, as shown in Figure 9.

Based on Figure 8 and Figure 9, the following conclusions can be drawn:

1. The parameter $\sqrt{A}$ exhibits an overall linear trend, with step changes occurring at specific values. The step periods vary among different satellites. These changes can be attributed to orbital maneuvers conducted by the satellite’s control system to maintain the satellite in its predetermined orbit or to respond to external perturbations.
2. The parameters *e* and $i_{0}$ exhibit wave-like increases or decreases within various intervals.
3. The parameters ω and Ω exhibit similar variation patterns, oscillating within the range of −π to π and undergoing abrupt changes when approaching the threshold. The parameter Ω exhibits two jumps with an interval of approximately 1 year and shows a stepwise decrease within an annual cycle, with each step lasting about 7 days. This is highly likely due to the influence of the Earth’s non-spherical gravitational field (Earth’s equatorial bulge effect), resulting in a gradual precession of the orbital plane and a stepwise decrease.
4. The parameter $M_{0}$ exhibits a linear increase over short periods (approximately 0.5 days) and an overall increase over long periods (approximately 1 month), with significant non-linear jumps occurring near ±π. Its jump pattern is similar to that of $\sqrt{A}$ but is also partially attributed to the sensitivity of the perigee and apogee locations.

Additionally, parameters Δn, $\dot{\Omega }$, IDOT, $C_{rc}$, $C_{rs}$, $C_{uc}$, and $C_{us}$ exhibit periodic variations with a primary period of approximately 0.5 years, mainly due to periodic perturbations. The parameters $C_{ic}$ and $C_{is}$ follow a period of roughly 16 days.

Moreover, the time-series data from MEO and IGSO satellites display a high level of consistency and similar trends, indicating that they can be grouped together for discussion.

### 3.4. Data Integrity Assessment

Building on the previous analysis, this study introduces a URA-based filtering mechanism during the data processing phase to mitigate spurious anomalies that could compromise the accuracy of the analysis. This approach ensures the generation of long-term, reliable, and stable ephemeris data, as summarized in Table 2.

In BDS2, the maximum missing value rate for satellite C3 reached 23.7%, whereas for BDS3, the maximum missing value rate for satellite C35 was 25.5%. The average data availabilities for BDS2 and BDS3 were 85.86% and 87.14%, respectively. The analysis reveals that the average availability of BDS3 satellites is comparatively higher, positively influencing the system’s stability and accuracy. This trend indicates significant progress in the optimization of the BDS3 system.

### 3.5. Stationarity Test

**Figure 10:**
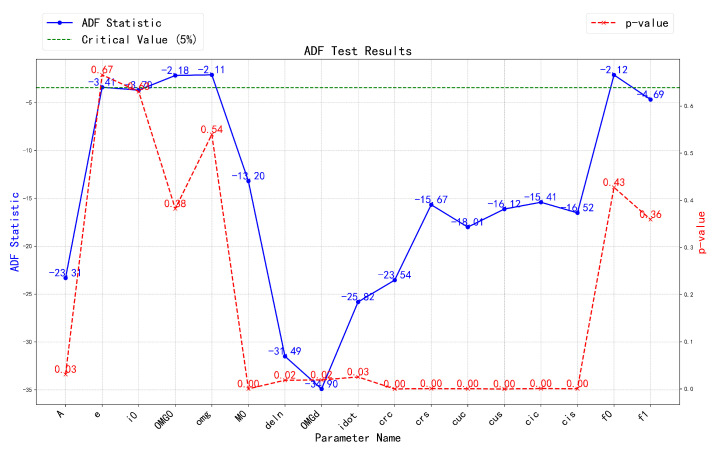
BDS broadcast ephemeris stability test results.

To assess the stationarity of the broadcast ephemeris data, the Augmented Dickey–Fuller (ADF) test was employed. The ADF test is a widely used method for detecting the presence of a unit root in a time series, offering insights into whether the series is stationary or influenced by trends and seasonality. In Figure 10, the blue solid line represents the ADF statistic, which indicates the degree of stationarity in the series. The red dashed line corresponds to the *p*-value, determining the significance of the test results, while the green dashed line represents the critical value, serving as a threshold for evaluating stationarity. These statistical indicators inform subsequent decisions during the data preprocessing phase.

As shown in Figure 10 above, for BDS broadcast ephemeris parameters $\sqrt{A}$, $M_{0}$, Δn, $\dot{\Omega }$, IDOT, $C_{rc}$, and $C_{rs}$, the ADF statistic is significantly below the critical value, with a mean *p*-value of 0.01 and a maximum of 0.03, indicating that these time series are stationary.

In contrast, for parameters *e*, $i_{0}$, Ω, and ω, the ADF statistic is above the critical value, and the *p*-value exhibits unstable wave-like behavior, suggesting that the significance is insufficient to reject the null hypothesis. This may be attributed to differences in the signal bands of various satellites. Additionally, while the ADF statistics for the orbital parameters $C_{uc}$, $C_{us}$, $C_{ic}$, and $C_{is}$ are −18.01, −16.12, −15.41, and −16.52 respectively—higher than those of the previously mentioned stationary parameters—their *p*-values are all 0, confirming their stationarity, albeit with relatively weaker stationarity.

Finally, for the clock parameters, specifically the clock bias $af_{0}$, clock drift $af_{1}$, and clock drift rate $af_{2}$ (where $af_{2}$ is 0 in the BDS parameter system and thus not involved in subsequent experiments), the ADF statistic values for $af_{0}$ and $af_{1}$ exceed the critical value, indicating they may be non-stationary time series. Furthermore, with *p*-values of 0.43 and 0.36, respectively, there is a high probability of a unit root, reinforcing the likelihood of non-stationarity. To address this issue, this study applies differencing to the data, transforming non-stationary time series into stationary sequences.

### 3.6. Time-Series Difference Processing

To enhance the role of broadcast ephemeris parameters from $C_{2}$ to $C_{19}$ as a supplementary technique in anomaly detection, this study establishes detection thresholds by applying first- or higher-order differencing to all mentioned parameters. This approach not only facilitates the identification of changes in the data but also provides a clearer basis for anomaly detection. The differenced data are subsequently fitted to distribution models, resulting in more intuitive and precise visualizations of time-series plots and histograms.

Feature analysis reveals that parameters Δn, $\dot{\Omega }$, IDOT, $C_{rc}$, $C_{rs}$, $C_{uc}$, $C_{us}$, $C_{ic}$, and $C_{is}$ exhibit similar trends within a single satellite. Thus, a selective set of parameters is presented in this study based on their significance in detecting anomalies.

**Figure 11:**
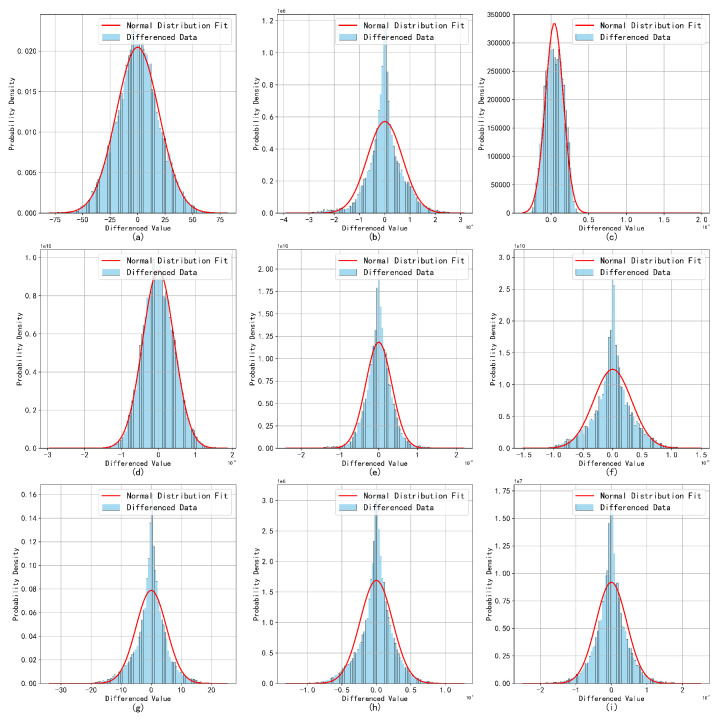
Broadcast ephemeris time-series difference distribution fitting results. As shown in the figure, subplots (**a**–**i**) respectively represent the normal distribution curves of the nine parameters, which correspond to Δn, $\dot{\Omega }$, IDOT, $C_{rc}$, $C_{rs}$, $C_{uc}$, $C_{us}$, $C_{ic}$, and $C_{is}$.

Figure 11 illustrates the corresponding normal distribution curves. It is important to note that the parameters Ω, ω, and $M_{0}$ are excluded from the analysis. This decision is based on the fact that these parameters exhibit highly stable periodic fluctuations across most satellites, making the analysis of incremental changes between consecutive observations less likely to yield meaningful insights.

The differencing of the clock bias sequence significantly enhances its stationarity. With the exception of the clock bias, the other parameters remain largely unchanged. The first-differenced sequences of the BDS broadcast ephemeris parameters approximate a normal distribution, indicating that only the clock bias requires differencing. The other parameters exhibit minimal fluctuations, and due to the inherent characteristics of the orbital parameters, it is more reasonable to retain the original data without diminishing trends. This conclusion is consistent with the results of the ADF test.

The fitting results for the normal distribution of the ephemeris parameters overall approach normality. However, certain parameters, such as $C_{is}$, exhibit outliers and abrupt changes that significantly impact the overall variance, leading to increased fitting standard deviations, wider distribution curves, and a slight tendency toward multimodality. This suggests the presence of multiple subgroups or anomalous fluctuations within the data.

**Figure 12:**
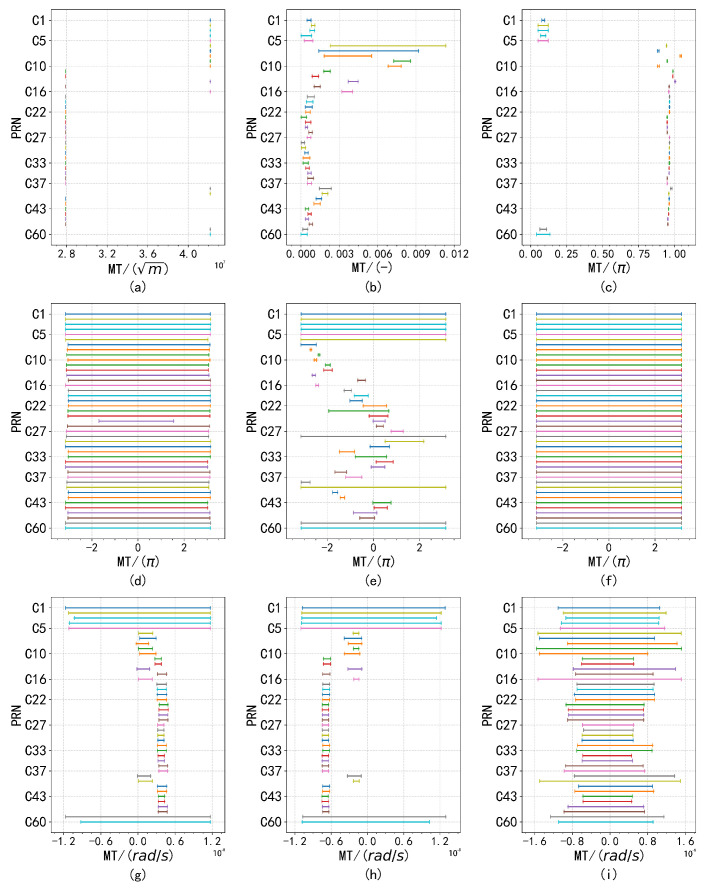
The figure demonstrates the monitoring thresholds of $\sqrt{A}$, *e*, $i_{0}$, Ω, ω, $M_{0}$, Δn, $\dot{\Omega }$, and IDOT in subplots (**a**–**i**). In Figure 12, Figure 13 and Figure 14 presented within the text, ‘MT’ refers to the monitoring threshold.

**Figure 13:**
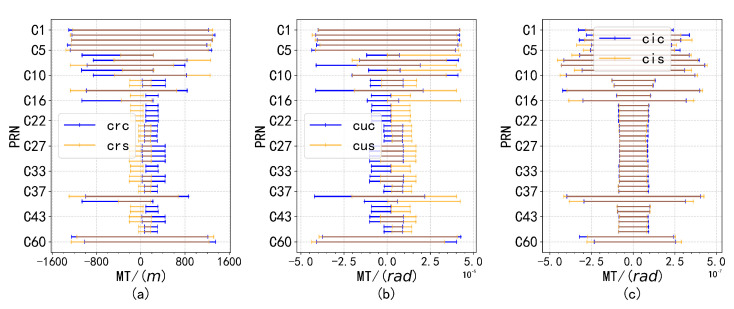
Subplots (**a**–**c**) show the distribution of differences for different parameter pairs, with $C_{rc}$, $C_{rs}$, $C_{uc}$, $C_{us}$, and $C_{ic}$, $C_{is}$ paired according to their respective threshold ranges.

**Figure 14:**
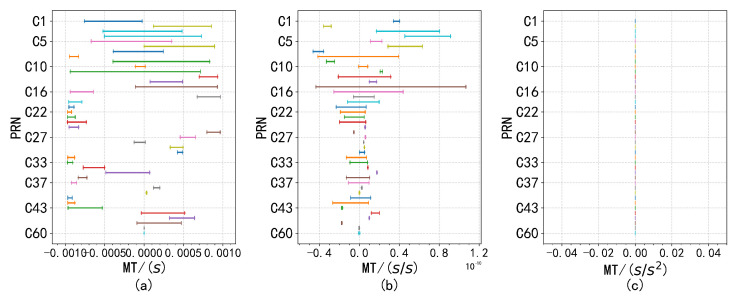
The monitoring thresholds for $f_{0}$, $f_{1}$ and $f_{2}$ are shown in subfigures (**a**–**c**), respectively, with $f_{2}$ remaining constant at 0.

Despite the presence of anomalous factors, the overall distribution exhibits normal characteristics. Analysis of data from other satellites corroborates this conclusion, as similar waveform patterns were observed. This finding suggests that while the presence of outliers may cause deviations from an ideal normal distribution, the overall normality remains intact, which is essential for accurate parameter estimation and analysis. Consequently, a monitoring threshold was defined, restricting the proportion of outlier data to no more than $10^{-4}$, as shown in Figure 12, Figure 13 and Figure 14.

To enhance the accuracy and reliability of data processing, this study performs a statistical analysis of the parameters $\sqrt{A}$, *e*, $i_{0}$, Ω, ω, and $M_{0}$ from the 2021 BDS broadcast ephemeris data. Preliminary observations of the broadcast ephemeris satellite data reveal discrepancies between the parameter values of different satellite types and the valid ranges specified in IS-GPS-200N [37].

Therefore, this study combines the differencing results with the IS-GPS-200N document to establish the valid ranges for each parameter, as shown in Table 3.

### 3.7. Correlation Analysis

**Figure 15:**
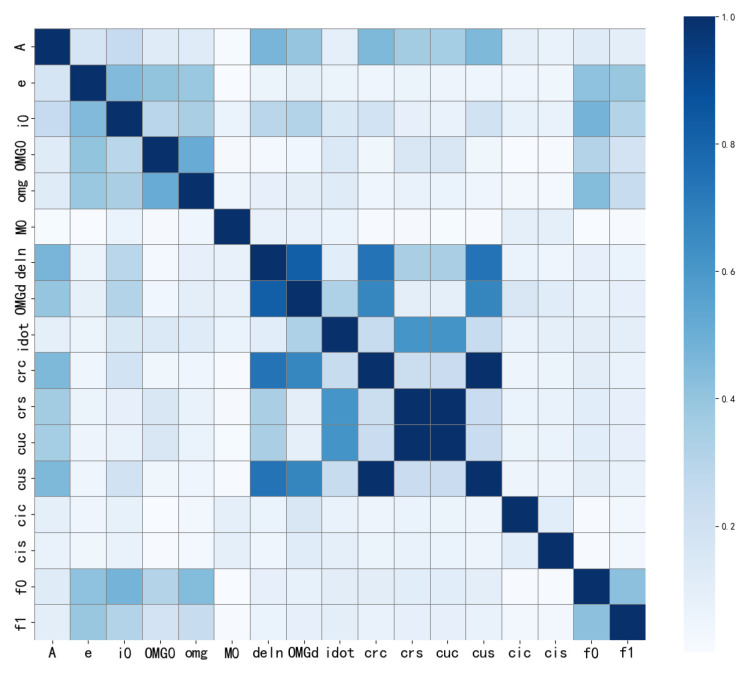
Correlation analysis of BDS broadcast ephemeris parameters.

A correlation analysis was conducted on the data to obtain the corresponding results, as illustrated in Figure 15. The findings align well with our expectations, confirming the relationships between the various parameters under study.

Figure 15 displays the average absolute correlation among the selected 17 BDS broadcast ephemeris parameters, with darker colors indicating stronger correlations and lighter colors indicating weaker ones. From the figure, it is evident that some parameters exhibit a high degree of correlation, while others show relatively weak correlations, as summarized in the following points:

1. $C_{uc}$ and $C_{rs}$ exhibit near-perfect correlation, indicating a high degree of consistency in their changing trends. Similarly, $C_{rc}$ and $C_{us}$ display the same characteristics. This analysis concludes that these pairs of parameters possess similar physical significance within the context of orbital characteristics and are influenced by the same factors, as reflected in their data similarity.
2. $\dot{\Omega }$ and IDOT demonstrate a moderate level of correlation, indicating a relationship that is weaker than that observed between $C_{uc}$ and $C_{rs}$. In contrast, $af_{0}$ and $af_{1}$ adhere to a fitted model; although their correlation is not significant, this fitting relationship underscores the importance of considering physical models during analysis rather than relying solely on correlation analysis.
3. Many parameters, such as $\sqrt{A}$, *e*, and $i_{0}$, exhibit weak correlations. In anomaly detection, absolute value analysis provides initial guidance; however, further investigation using physical fitting models is necessary to ensure that potential relationships between key parameters are not overlooked.

## 4. Results

### 4.1. BDS Broadcast Ephemeris Orbit Parameter Prediction

The subset of data was processed using methods such as differencing. As a predictive multivariate time-series anomaly detection model, the broadcast ephemeris data, excluding specific parameters like satellite clock bias $af_{1}$ and health indicators, predominantly display periodic time-series characteristics. This study integrated the findings from the literature and the evaluation results to forecast parameters, including Δn, $\dot{\Omega }$, IDOT, $C_{ic}$, $C_{is}$, and clock bias. Due to the systemic relationships among the internal parameters of the broadcast ephemeris data, four parameters—$\sqrt{A}$, $i_{0}$, Ω, and $af_{1}$—were included as related variables in the prediction process.

The prediction ranges were determined by capturing short- to medium-term trends and periodicity, specifically involving 7-step forecasts based on 22 steps, 10-step forecasts based on 40 steps, and 25-step forecasts based on 110 steps. With the exception of clock bias and $\dot{\Omega }$, all other parameters met the aforementioned criteria, where epochs ranged from 40 to 120. The interdependencies among parameters allowed this study to infer positional and temporal corrections through orbital mechanics and time-adjustment formulas.

**Figure 16:**
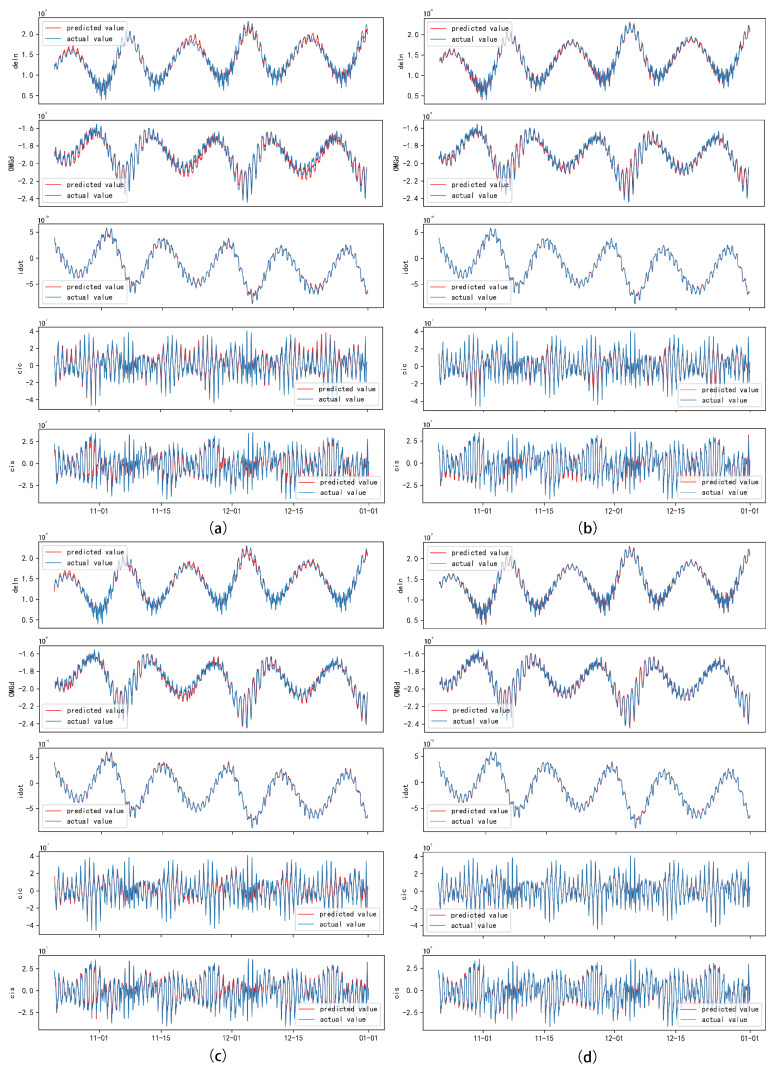
Comparisonof predicted orbital parameter results with actual measurements for selected satellites. The red line indicates predicted values, while the blue line indicates actual values. The dataset has been processed for outlier detection using robust Methods and iForest. Subfigure (**a**) represents LSTM, subfigure (**b**) represents A-LSTM, subfigure (**c**) represents TE-LSTM, and subfigure (**d**) represents IF-TEA-LSTM.

To forecast the broadcast ephemeris time series, a sliding-window strategy was employed, dividing the training and testing sets into several sliding time windows *T*. The overall loss function was defined as the root mean square error (RMSE) between the true values and predicted values for future time windows. The error metrics displayed in the figures indicate excellent performance in the context of broadcast ephemeris predictions. Selected error results are presented in the accompanying figure (Figure 16).

An analysis of the results indicated that for parameters such as ($\dot{\Omega }$) and (Δn), the LSTM exhibited a lag in predicted values relative to the actual data within certain intervals. Longitudinal observations revealed that this lag primarily occurred during the descending phases of the data trends. We hypothesize that the sliding time window strategy resulted in a delayed response of the model when faced with rapidly changing trends. To address this issue, we introduced an attention mechanism and a time-embedding mechanism to extract richer temporal features, significantly enhancing the model’s predictive accuracy during periods of decline.

Table 4 presents the average prediction errors for the orbital parameters. Initially, the LSTM model demonstrated strong performance in processing orbital parameters with significant volatility and periodic patterns, achieving satisfactory prediction accuracy. The incorporation of the attention mechanism further enhanced the model’s predictive capabilities, significantly improving the fit between the predicted values and the actual measurements, with error rates consistently maintained below 10%. Additionally, the introduction of threshold control methods optimized the model’s predictive accuracy.

**Figure 17:**
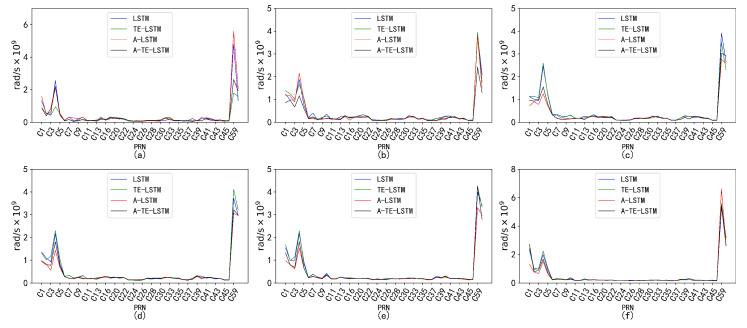
Prediction accuracy and performance of Δn under different reference frames. The six subplots (**a**–**f**) represent this parameter under the following forecast time horizons: (**a**) 24 h forecast, 1 h interval; (**b**) 96 h forecast, 1 h interval; (**c**) 7 d forecast, 1 h interval; (**d**) 15 d forecast, 1 h interval; (**e**) 30 d forecast, 1 h interval; (**f**) 90 d forecast, 1 h interval. The subplot distribution in Figure 18, Figure 19, Figure 20 and Figure 21 follows the same structure.

An analysis of the RMSE values for four models—LSTM, A-LSTM, TE-LSTM, and IF-TEA-LSTM—revealed their performance, as shown in Figure 17. The LSTM model struggled to capture the nonlinear variations in BDS satellite orbital parameters, particularly for Δn, which is influenced by external factors such as multipath effects and space weather, resulting in relatively high RMSE values. However, the integration of the attention mechanism and time-embedding mechanism significantly improved feature extraction in the A-TE-LSTM model, leading to notable improvements in prediction accuracy.

The performance of Δn varied across different time intervals when predicted using the TE-LSTM, A-LSTM, and A-TE-LSTM models. The RMSE values for TE-LSTM consistently improved over LSTM, ranging from 9.4% to 10.5%. In contrast, A-LSTM exhibited greater fluctuations, decreasing by 4.37% in the 24-hour forecast and achieving a maximum improvement of 20.92% in longer-term predictions. Notably, A-TE-LSTM demonstrated the most significant performance, with enhancements ranging from 12.61% to 25.06%, particularly evident in long-term forecasting scenarios.

In the prediction of the IDOT parameter, all four models demonstrated commendable performance. The TE-LSTM model showed an average improvement ranging from 7.18% to 13.42%, indicating a stable enhancement effect. The A-LSTM model also exhibited notable gains, with a maximum increase of 22.46%. However, the A-TE-LSTM model stood out overall, achieving improvements between 21.90% and 27.29%, further validating its superiority in feature extraction and accuracy. Overall, all three models performed admirably in predicting the IDOT parameter.

The performance of the models in predicting the $\dot{\Omega }$ parameter varied significantly across different time intervals. The TE-LSTM model demonstrated an average enhancement ranging from 6.90% to 15.81%. The A-LSTM model also showed substantial improvements, achieving a maximum increase of 25.92%. Notably, the A-TE-LSTM model excelled, achieving an overall enhancement between 19.71% and 38.39%, particularly remarkable in short-term forecasts (24 h and 96 h), thereby showcasing its robust capability for feature extraction. The pronounced differences in predictive performance across various time periods can be attributed to the influence of nonlinear errors, such as multipath effects, which significantly impacted the $\dot{\Omega }$ parameter. These error sources can accumulate over time, severely affecting overall prediction accuracy.

The parameters $C_{is}$ and $C_{ic}$ exhibited minimal correction effects and were less influenced by external disturbances, resulting in relatively low variability. Consequently, the performance of the TE-LSTM, A-LSTM, and A-TE-LSTM models showed significant discrepancies. For $C_{is}$, the TE-LSTM model demonstrated suboptimal performance, with average improvements ranging from −6.26% to −0.60%, indicating poor predictive capability. The A-LSTM model exhibited stable performance in short-term predictions (24 h and 96 h), albeit with limited improvements; negative values were observed in some cases, such as 30-day and 90-day forecasts. The A-TE-LSTM model performed slightly better in certain scenarios, particularly in the 7-day and 15-day forecasts, achieving improvements of 7.22% and 6.12%, respectively. However, the overall enhancement remained constrained, likely due to frequent orbital adjustments of $C_{is}$ in the short term, which complicated the modeling of nonlinear errors.

The prediction results for $C_{ic}$ revealed poor performance in the TE-LSTM model’s 24-h forecast, with a negative improvement of −5.20%. However, in longer-term predictions, particularly for 7-day and 15-day forecasts, improvements reached as high as 6.28%. The A-LSTM model exhibited relatively stable performance, while the A-TE-LSTM model excelled, maintaining high improvement rates across all forecasts, especially in the 96-hour and 7-day predictions, with enhancements of 15.17% and 13.27%, respectively.

The presence of substantial noise in some parameters contributed to an overall increase in prediction errors. This noise manifested as random errors and irregular fluctuations; for instance, the RMSE metric for $C_{is}$ displayed significant volatility, indicating that noise considerably affected the model’s accuracy in later predictions. Even the IF-TEA-LSTM model struggled to fully capture these noise effects, complicating its ability to mitigate irregular errors and thus impacting overall predictive performance.

**Figure 22:**
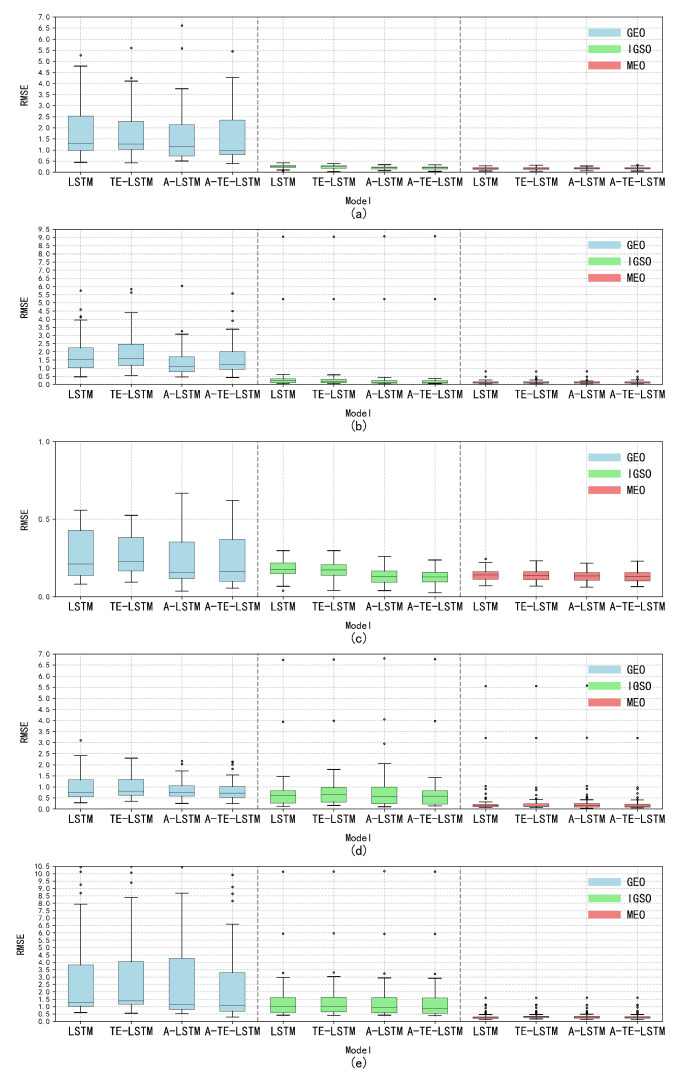
Analysis of the distribution characteristics of the five major anomaly parameters under different orbit types. Subplot (**a**) illustrates the distribution characteristics of the Δn parameter across GEO, IGSO, and MEO orbit types; subplot (**b**) shows the distribution of the $\dot{\Omega }$ parameter under the same orbit types; subplot (**c**) depicts the distribution characteristics of the IDOT parameter for GEO, IGSO, and MEO; subplot (**d**) highlights the distribution of the $C_{ic}$ parameter across the three orbit types; and subplot (**e**) presents the distribution characteristics of the $C_{is}$ parameter for GEO, IGSO, and MEO.

Through an in-depth analysis of the RMSE, we observed significant characteristic differences among satellite types. The GEO-type LSTM model exhibited consistently high RMSE values, indicating considerable errors in processing GEO orbital data. In contrast, IGSO and MEO types excelled in error control, showcasing lower RMSE values and tighter distributions. Notably, certain models demonstrated superior performance for specific orbit types, particularly the A-TE-LSTM model, which consistently achieved lower RMSE values, highlighting its potential advantages in particular application scenarios, as illustrated in Figure 22.

Therefore, it is crucial to distinguish between different orbit types, with particular emphasis on the classification of GEO orbital types, in order to mitigate substantial errors typically associated with these types compared to others. The primary objective of this study is anomaly detection, where RMSE accuracy and the presence of minor abrupt anomalies do not significantly affect the overall detection performance.

Considering that the model processes multi-parameter, multi-variable time-series data with high correlation among parameters, anomalies can still be effectively identified by analyzing trends and fluctuations. For example, while parameters such as $C_{ic}$ and $C_{is}$ may exhibit short-term volatility, a joint analysis of these parameters can uncover underlying anomaly patterns, thus enhancing the accuracy of detection.

### 4.2. BDS Broadcast Ephemeris Clock Parameter Prediction

The clock bias is treated separately due to its inherent complexity, which distinguishes it from the orbital parameters. The performance of the LSTM model on raw clock bias data was found to be limited, leading to poor fitting results. To address this issue, a stabilization process was applied to the clock bias data, incorporating outlier detection techniques to identify anomalies. Following this, segmented linear interpolation was utilized to replace the anomalous data points. Predictive experiments conducted on the refined dataset demonstrated the effectiveness of this approach, significantly improving the accuracy of clock bias predictions.

**Figure 23:**
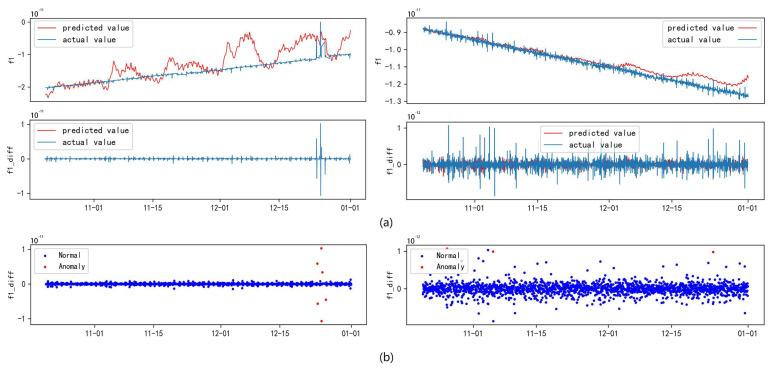
Comparison chart of clock bias parameter prediction results and actual results for some satellites. Subplot (**a**) represents the comparison between the prediction results before and after differencing, while subplot (**b**) represents the anomalies detected by IF-TEA-LSTM.

Figure 23 presents a comparison between the actual and predicted values of the broadcast ephemeris clock bias sequence. In theory, if the accuracy of the actual values exceeds 99.5%, the predicted values should closely align with them, forming a nearly smooth diagonal line. This alignment would indicate the model’s capability to effectively capture the fluctuations of non-stationary sequences, such as clock bias. However, traditional time-series models like LSTM often struggle to accurately fit such complex nonlinear sequences, resulting in diminished prediction performance.

To address this challenge, second-order differencing was applied to the clock bias sequence, reducing trend-related interference and allowing the model to extract more meaningful information. As shown in subplot (b) of Figure 23, the predictions after differencing reveal significant deviations from the ideal diagonal line, highlighting anomalous timestamps.

Additionally, specific experimental satellites were selected for model validation using representative samples. The RMSE values in Table 5 further demonstrate the LSTM model’s performance, particularly noting the improvements after differencing. Although no significant anomalies were detected in the sample, minor deviations were observed in the model’s predictions. These deviations remained within acceptable limits, reinforcing the conclusion that differencing preprocessing enhances the predictive accuracy of complex non-stationary sequences. These results emphasize the crucial role of data preprocessing in improving the performance of time-series prediction models.

As shown in the table, the IF-TEA-LSTM model consistently outperformed other models in clock bias prediction and anomaly detection. The following points summarize its performance:

1. For satellites C21 and C22, within a 96-hour short-term forecast window, the RMSE of the IF-TEA-LSTM model improved by 62.56% and 63.52% compared to the standard LSTM, 36.82% and 34.62% compared to A-LSTM, and 60.70% and 63.45% compared to TE-LSTM.
2. For satellites C26 and C27, the IF-TEA-LSTM model showed RMSE improvements of 69.75% and 77.61% over LSTM, 52.12% and 44.91% over A-LSTM, and 70.38% and 76.75% over TE-LSTM.

It is important to note that the MAE, RMSE, MSE, and range values in the table were derived from normalized and differenced data. Therefore, the units differ from those of the raw data (e.g., clock bias measured in seconds, refined to nanoseconds). These metrics were used for the relative model performance comparison and do not represent the final absolute errors. Upon denormalization to the original scale, the error metrics will revert to units consistent with the raw data.

### 4.3. Performance of BDS Broadcast Ephemeris Data Anomaly Detection

We analyzed the broadcast ephemeris data of BDS2 and BDS3 satellites, focusing on the performance differences across different orbits (GEO, IGSO, MEO) using an RNN, a GRU, LSTM, and their enhanced versions. The experimental results demonstrate that LSTM outperformed the RNN and GRU in time-series modeling, showing superior precision, recall, and F1-score. Although the GRU offers improved computational efficiency, its recall rate was relatively lower when handling long-term broadcast ephemeris data, indicating its limitations in capturing long-term dependencies. Moreover, the GRU’s extended training time, without corresponding performance gains, suggests structural limitations in learning long sequences. The detailed results for each satellite type are shown in Table 6.

For the GEO orbital data, the IF-TEA-LSTM model demonstrated the best performance, achieving 86.43% precision and 74.57% recall, with A-LSTM and LSTM closely following. This indicates that more complex models have a clear advantage in capturing the temporal features of GEO data, particularly in identifying anomalies. In contrast, the GRU and RNN models exhibited lower recall, revealing their limitations in handling GEO data.

For the IGSO data, the IF-TEA-LSTM model continued to excel, with precision reaching 89.64% and recall at 79.62%. A-LSTM and LSTM also performed well in this orbit, especially in terms of precision and F1-score, underscoring the effectiveness and adaptability of LSTM-based models in processing dynamic data from non-synchronous orbits.

In the MEO orbital experiments, IF-TEA-LSTM maintained its lead, with precision at 93.42% and an F1-score of 89.21%, demonstrating strong generalization capabilities in processing medium Earth orbit satellite data. A-LSTM and TE-LSTM also showed robustness, while the GRU and RNN models continued to struggle in terms of recall, reflecting their inability to capture the complex temporal patterns of MEO data.

The results indicate that orbital conditions and data characteristics significantly influence model performance. GEO satellites, due to their large static range and limited dynamic properties, underperformed compared to IGSO and MEO satellites. This highlights the need for more specialized methods when analyzing GEO data. In contrast, the higher dynamism in IGSO and MEO data poses challenges for machine learning models, but with extensive training data (e.g., over 1–3 years, exceeding 1.15 million records), models exhibit strong adaptability and predictive power.

Notably, the TE-LSTM model improved average performance by 4.31% for GEO orbits, while the improvements for IGSO and MEO orbits were 0.878% and 2.16%, respectively. This suggests that the sensitivity of TE-LSTM to threshold constraints is more pronounced for GEO data, where stronger threshold enforcement significantly enhances anomaly detection. For other orbits, the effect is relatively smaller. Therefore, optimizing threshold constraints for different orbit types is crucial for improving anomaly detection accuracy. Table 7 summarizes the average performance of various models across different orbits, further validating these findings.

The overall experimental results indicate that the proposed IF-TEA-LSTM model demonstrates significant advantages across various evaluation metrics. This finding validates the effectiveness of the methods introduced in this study, showcasing the model’s strong generalization capabilities and precise predictive power. By deeply learning temporal features, the model effectively identifies and captures hidden anomaly patterns, providing a broadly applicable and efficient reference framework for future broadcast ephemeris data processing and anomaly detection.

Furthermore, by integrating a clock bias scoring mechanism, the IF-TEA-LSTM model enhances its anomaly detection capabilities in practical applications. Given the critical role of clock bias data in satellite navigation systems, the introduction of this scoring mechanism not only improves the model’s ability to identify anomalous data points in the broadcast ephemeris but also allows for more accurate localization and classification of potential anomalies. This provides robust technical support for the health monitoring and subsequent maintenance of satellite orbital data. Therefore, the series of innovative improvements based on LSTM and its derivatives not only enhances anomaly detection performance across various orbital data but also lays a solid theoretical and practical foundation for future processing and precise prediction of more complex, multidimensional ephemeris data.

## 5. Conclusions

This study proposes a robust anomaly detection method based on broadcast ephemeris data, aimed at improving the real-time safety and reliability of satellite operations. This approach converts broadcast ephemeris data into a multivariate time series and applies the IF-TEA-LSTM model, integrating diverse evaluation techniques to enhance anomaly detection. Initial analyses revealed that raw broadcast ephemeris data alone are insufficient for precise anomaly identification. To overcome this limitation, this study incorporates detailed evaluations of thresholds, distributions, and temporal patterns. The processed data, combined with the enhanced IF-TEA-LSTM model, demonstrate the model’s effectiveness in anomaly detection while uncovering distribution characteristics across different satellite types. Both the baseline LSTM and the improved IF-TEA-LSTM models, supported by SIS and iForest techniques, show promising results in ensuring satellite operation reliability.

In terms of predictive accuracy, the IF-TEA-LSTM model outperformed other methods across all parameters. It achieved MAE, RMSE, and MSE values of 0.2156, 0.2387, and 0.0570. Specific parameters like Δn and $\dot{\Omega }$ saw RMSE reductions of 25.06% and 38.39%, respectively, while IDOT improved within a range of 21.90% to 27.29%. Although $C_{is}$ and $C_{ic}$ were more susceptible to noise, certain time periods still yielded performance gains of up to 13.27%. Notably, the prediction errors for GEO orbits were significantly higher than those for IGSO and MEO orbits, underscoring the importance of orbit type classification in enhancing prediction accuracy. For clock bias prediction, using four representative MEO satellites, the model achieved a 68.37% improvement over baseline models and a 42.62% enhancement compared to the A-LSTM model over a 96-hour short-term forecast.

This comprehensive analysis highlights the dual capability of the IF-TEA-LSTM model in both accurate prediction and effective anomaly detection, showcasing its potential for improving satellite operation monitoring and reliability across diverse scenarios.

## Figures and Tables

**Figure 18:**
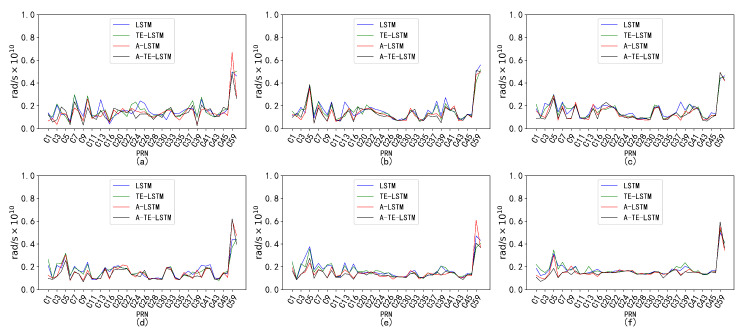
Prediction accuracy and performance of IDOT under different reference frames. The six subplots (**a**–**f**) show the parameter for forecast periods of 24 h, 96 h, 7 days, 15 days, 30 days, and 90 days, each with a 1-h interval.

**Figure 19:**
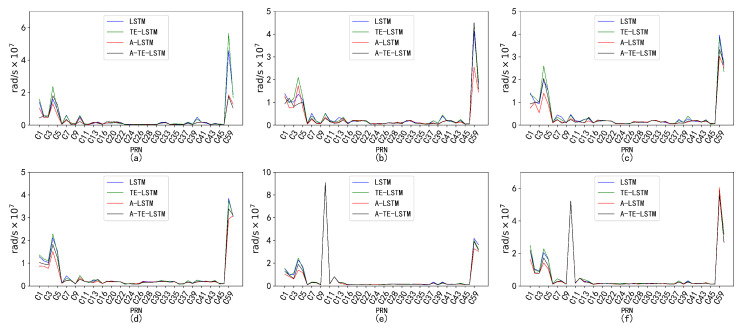
Prediction accuracy and performance of $\dot{\Omega }$ under different reference frames. The six subplots (**a**–**f**) show the parameter for forecast periods of 24 h, 96 h, 7 days, 15 days, 30 days, and 90 days, each with a 1-hour interval.

**Figure 20:**
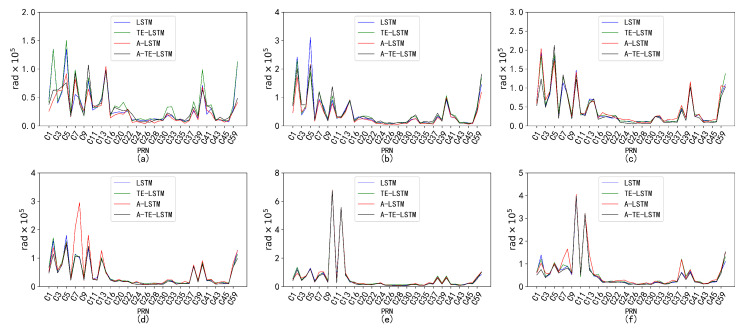
Prediction accuracy and performance of $C_{is}$ under different reference frames. The six subplots (**a**–**f**) show the parameter for forecast periods of 24 h, 96 h, 7 days, 15 days, 30 days, and 90 days, each with a 1-hour interval.

**Figure 21:**
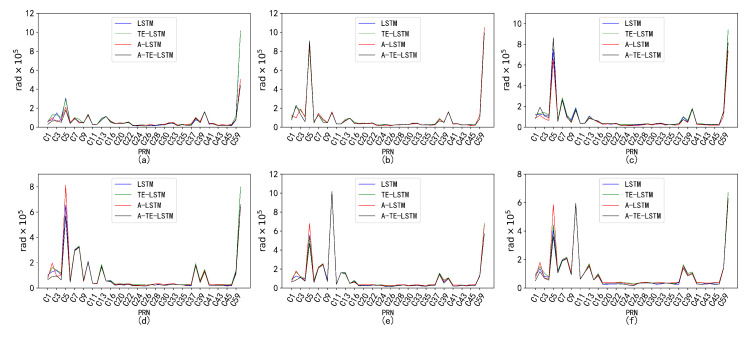
Prediction accuracy and performance of $C_{ic}$ under different reference frames. The six subplots (**a**–**f**) show the parameter for forecast periods of 24 h, 96 h, 7 days, 15 days, 30 days, and 90 days, each with a 1-hour interval.

**Table 1:** Satellite data acquisition source.

PRN	C1	C2	C3	C4	C5	C6	C7	C8	C9	C10	C11
	C12	C13	C14	C16	C19	C20	C21	C22	C23	C24	C25
	C26	C27	C28	C29	C30	C32	C33	C34	C35	C36	C37
	C38	C39	C40	C41	C42	C43	C44	C45	C46	C59	C60
Data Type	Broadcast ephemeris										
Time Span	From 31 December 2020 to 31 December 2021.										
Data Source	IGS Data Center of WHU										
FTP address	ftp://igs.gnsswhu.cn and http://www.igs.gnsswhu.cn/ (accessed on 18 October 2024)										
Data Expansion	CDDIS										
FTP address	ftp://gdc.cddis.eosdis.nasa.gov/archive/gnss/data (accessed on 18 October 2024)										

Ensure that the necessary permissions and tools are obtained to access the FTP server, access to the WHU server necessitates an application, whereas access to CDDIS is publicly available.

**Table 2:** Detailed table of BDS broadcast ephemeris data availability.

PRN	C1	C2	C3	C4	C5	C6	C7	C8	C9	C10	C11
Availability (%)	87.3	84.5	76.3	79.4	82.8	86.5	89.3	87.6	83.9	90.5	92
PRN	C12	C13	C14	C16	C19	C20	C21	C22	C23	C24	C25
Availability (%)	93.3	81.5	92.8	89.2	89.6	88.3	79.8	82.5	86.4	87.8	87.1
PRN	C26	C27	C28	C29	C30	C32	C33	C34	C35	C36	C37
Availability (%)	85.3	82.4	93.1	87.4	83.8	87.6	81.6	87.9	74.5	89.1	94.3
PRN	C38	C39	C40	C41	C42	C43	C44	C45	C46	C59	C60
Availability (%)	91.6	92.7	89.6	86.8	87.3	79.1	83.9	91.4	88.2	89.4	81.5

**Table 3:** Characteristics and thresholds of BDS broadcast ephemeris parameters.

Parameter	Data Name	Valid Range	Unit
$t_{oc}$	toc	-	−
$\sqrt{A}$	A	0∼8192	$m^{1/2}$
*e*	e	0∼0.02	−
$i_{0}$	i0	0∼1	π
Ω	OMG0	−1∼1	π
ω	omg	−1∼1	π
$M_{0}$	M0	−1∼1	π
Δn	deln	$-3.73\times 10^{-9}∼3.73\times 10^{-9}$	rad/s
$\dot{\Omega }$	OMGd	$-1.728\times 10^{-8}∼1.728\times 10^{-8}$	rad/s
IDOT	idot	$-9.31\times 10^{-10}∼9.31\times 10^{-10}$	rad/s
$C_{rc}$	crc	−1622∼1622	m
$C_{rs}$	crs	−1622∼1622	m
$C_{uc}$	cuc	$-5.23\times 10^{-5}∼5.23\times 10^{-5}$	rad
$C_{us}$	cus	$-5.23\times 10^{-5}∼5.23\times 10^{-5}$	rad
$C_{ic}$	cic	$-6.10\times 10^{-5}∼6.10\times 10^{-5}$	rad
$C_{is}$	cis	$-6.10\times 10^{-5}∼6.10\times 10^{-5}$	rad
$af_{0}$	f0	$-9.77\times 10^{-4}∼9.77\times 10^{-4}$	s
$af_{1}$	f1	$-3.73\times 10^{-9}∼3.73\times 10^{-9}$	s/s
$af_{2}$	f2	$-3.55\times 10^{-15}∼3.55\times 10^{-15}$	${\mathrm{s/s}}^{2}$

BDS relies on the GPS18 parameter model, with broadcast ephemeris data interpreted per the ICD’s $t_{oc}$ and $t_{oe}$, potentially impacting ephemeris parameters and model fitting. However, the anomaly detection research is fundamentally based on the integration of Keplerian orbital elements and perturbation terms, thus remaining unaffected.

**Table 4:** Average prediction error of BDS broadcast ephemeris orbit parameters.

Model	MAE	RMSE	MSE
LSTM	0.3357	0.5653	0.3196
A-LSTM	0.2775	0.2818	0.0794
TE-LSTM	0.3126	0.4242	0.1799
IF-TEA-LSTM	0.2156	0.2387	0.0570

**Table 5:** Prediction performance of clock bias parameters for satellites C21, C22, C26, and C27 under different models.

PRN	Error Metrics (s)	LSTM	TE-LSTM	A-LSTM	IF-TEA-LSTM
C21	MAE	0.1854	0.1253	0.1653	0.0857
	RMSE	0.6553	0.6243	0.3886	0.2455
	MSE	0.4294	0.3898	0.1510	0.0603
	Range	0.3452	0.3094	0.2287	0.1458
C22	MAE	0.1632	0.1143	0.1482	0.0759
	RMSE	0.3551	0.3544	0.1980	0.1295
	MSE	0.1261	0.1256	0.0392	0.0168
	Range	0.2194	0.1865	0.1559	0.0967
C26	MAE	0.1426	0.0967	0.1357	0.0653
	RMSE	0.2944	0.3008	0.1860	0.0891
	MSE	0.0867	0.0905	0.0346	0.0079
	Range	0.2048	0.1847	0.1342	0.0897
C27	MAE	0.1793	0.1295	0.1542	0.0721
	RMSE	0.5746	0.5539	0.2337	0.1287
	MSE	0.3302	0.3068	0.0546	0.0166
	Range	0.2982	0.2649	0.1794	0.1128

**Table 6:** Comparison of detection performance of different models on different orbit types.

Orbit	Metric	RNN	GRU	LSTM	A-LSTM	TE-LSTM	IF-TEA-LSTM
GEO	Precision (%)	75.34	71.45	76.48	84.79	81.65	86.43
	Recall (%)	62.12	53.22	64.74	72.85	64.47	74.57
	F1 (%)	67.52	61.36	69.31	78.62	72.43	79.23
IGSO	Precision (%)	81.24	76.33	82.92	88.76	85.22	89.64
	Recall (%)	68.75	56.25	71.14	78.85	71.42	79.62
	F1 (%)	74.54	65.07	76.80	83.67	77.48	84.51
MEO	Precision (%)	84.45	79.71	87.58	92.12	89.93	93.42
	Recall (%)	72.85	61.11	76.64	84.54	76.94	85.36
	F1 (%)	78.41	69.77	81.00	88.09	82.79	89.21

**Table 7:** Comparison of comprehensive performance metrics of different models on various orbit types.

Model	Precision (%)	Recall (%)	F1 (%)
RNN	80.34	67.91	73.49
GRU	75.83	56.86	65.40
LSTM	82.33	70.84	75.70
A-LSTM	88.56	78.75	83.46
TE-LSTM	85.60	70.94	77.57
IF-TEA-LSTM	89.83	79.85	84.32

## Data Availability

The original data used to obtain the results in this study can be accessed via the FTP or the official website http://www.igs.gnsswhu.cn/index.php (accessed on 1 January 2024), or by contacting the author via email at 222208855042@zust.edu.cn. The differential processing version of the data, organized datasets, RTKLIB data reading codes, and latest raw data from 2023 onward are currently being prepared; please feel free to reach out to me via email for further information.

## Abbreviations

The following abbreviations are used in this manuscript:

NASA	National Aeronautics and Space Administration
TEC	Total Electron Content
CDDIS	Crustal Dynamics Data Information System
RAIM	Receiver Autonomous Integrity Monitoring
GNSS	Global Navigation Satellite System
PNT	Positioning, Navigation, and Timing
BDS	BeiDou Navigation Satellite System
IGSO	Inclined Geosynchronous Orbit
SISRE	Signal-in-Space Range Error
RNN	Recurrent Neural Network
LSTM	Long Short-Term Memory
ADF	Augmented Dickey-Fuller
RMSE	Root Mean Square Error
SVM	Support Vector Machine
SIS	Signal-in-Space
GRU	Gated Recurrent Unit
iForest	Isolation Forest
MAE	Mean Absolute Error
URA	User Range Accuracy
GEO	Geostationary Orbit
MEO	Medium Earth Orbit
MSE	Mean Squared Error
OMC	observation matrix
LEO	Low Earth Orbit
TE	Time Embedding
TOC	Time of Clock
DT	Decision Tree
RF	Random Forest
